# Longitudinal analysis of occupational prestige in Switzerland, 1946–2023: navigating economic modernization and changing labor market conditions

**DOI:** 10.3389/fsoc.2025.1570326

**Published:** 2025-06-25

**Authors:** Richard Nennstiel, Rolf Becker

**Affiliations:** Department of Sociology of Education, Institute of Educational Science, University of Bern, Bern, Switzerland

**Keywords:** occupational career, prestige, economic modernization, growth curve model, Swiss Household Panel

## Abstract

This study examines the long-term impact of educational expansion, occupational restructuring, and economic modernization on the prestige trajectories of Swiss men and women from 1946 to 2023. The impacts of these macro trends on individuals' career prospects are discussed by applying different theoretical approaches used in labor market research, such as the human capital approach, signal and filter theory, the labor queue model, the vacancy competition model, as well as the theory of labor market segmentation. Using data from the Swiss Household Panel and historical macroeconomic indicators, we apply growth curve models to analyze how structural changes shape occupational prestige over the life course and we test several hypotheses derived from different theoretical approaches (e.g., human capital theory, signal and filter theory, skill-biased technological change, and the vacancy competition model). Our findings reveal significant differences between cohorts, with younger generations benefiting from educational expansion and the shift toward a service economy. While modernization and labor market conditions influence career entry prestige, the role of education has become increasingly decisive over time, mitigating adverse structural effects. Men's prestige trajectories are more sensitive to macroeconomic fluctuations, while women's career advancements are more strongly linked to educational investment. Cohort size effects indicate increased intra-cohort competition, particularly among men. The study highlights the interplay between individual qualifications and structural labor market constraints, emphasizing the importance of a dynamic micro-macro approach for understanding social mobility. These findings contribute to the broader discourse on occupational stratification and the long-term returns to education, in the context of modernizing labor markets.

## Introduction

Like all other countries, Switzerland experiences constant social change, as concerns its social stratification and class structure (Bergmann et al., 2002; Jann and Combet, 2012; Becker and Jann, 2017; Falcon, 2020). In terms of both the system integration and social integration, change in the level of social inequality occurs at a more moderate pace in Switzerland than in other Western countries (Tillmann et al., 2018). In common with other Western countries, in Switzerland, social change—in regard to educational trajectories and occupational careers—occurs across consecutive birth cohorts, as Ryder (1965) described (Oesch, 2006, 2013; Buchmann et al., 2007; Hadjar and Berger, 2010; Jann and Combet, 2012; Becker and Zangger, 2013; Zangger and Becker, 2016; Nennstiel and Becker, 2023). Because they are influenced in different ways by societal conditions and by historical events, each birth cohort differs systematically from those cohorts born before or after them in their expectations, their decisions, their actions, and the resulting consequences for their life course (Becker and Blossfeld, 2017; Becker and Mayer, 2019; Becker et al., 2024). In this respect, consecutive birth cohorts are the cultural carriers of social change (Mayer and Huinink, 1990).

Educational expansion in Switzerland provides an obvious example of cohort differentiation relating to social change (Buchmann et al., 1993). In addition to the intergenerational reproduction of education, several empirical studies have shown that there has been an increase in the level of participation in higher education across consecutive birth cohorts in Switzerland since the 1950s (Buchmann et al., 2007; Hadjar and Berger, 2010; Jann and Combet, 2012; Becker and Zangger, 2013; Zangger and Becker, 2016). Although educational expansion was rather hesitant until the early 1980s, it accelerated for younger birth cohorts born after 1970 (Becker and Nennstiel, 2024, p. 39). Upward educational mobility resulted in qualificational upgrading within the parental generation and in offspring generations (Nennstiel and Becker, 2023). Another example of social change across birth cohorts has been the sectoral shift, occupational change, and transformation in job structures that has taken place in Switzerland since the Second World War (Sacchi et al., 2005; Oesch, 2013). Since the 1970s in particular, the economic focus has shifted from agricultural and industrial production to service activities (Oesch, 2006). According to the Swiss Office of Statistics (FSO), in 2023 some 77.5% (1970: 56%) of the workforce in Switzerland worked in the service sector (tertiary sector), 20.2% (1970: 35%) worked in the industrial sector (secondary sector), and 2.3% (1970: 9%) worked in agriculture (primary sector) (Sheldon, 2005, p. 25). For younger cohorts, this development has been interrelated with educational expansion across cohorts, as well as with technological change across periods. On the one hand, there is empirical evidence that the qualificational upgrading in the Swiss population resulted in occupational upgrading (Oesch and Menés, 2011, p. 527). On the other hand, it cannot be ruled out that tertiarization and the increased qualification level of professional activities have further fueled educational expansion (Goldin and Katz, 2009; Oesch and Menés, 2011; Acemoglu and Autor, 2012; Oesch, 2013; Becker and Blossfeld, 2022). Intergenerational mobility processes might illustrate cohort differentiation in regard to rewards for previous investments in education (Jann and Combet, 2012; Falcon, 2020). While educational expansion has resulted in an increase in upward mobility across generations, i.e., in increased returns to education in the course of occupational upgrading across birth cohorts, the structure of social mobility has not changed substantially (Oesch, 2006; Falcon, 2020).

There is a wealth of research on intragenerational occupational career mobility in various contexts (e.g., for the UK: Fellini and Guetto, 2019; Block and Jonsson, 2025; for South Korea: Kye et al., 2022; for the US: Cheng and Park, 2020; for Germany: Lersch et al., 2020; for France: Veljkovic, 2021; for Sweden: Bihagen et al., 2024; for a review article on current intragenerational mobility research, see also Cardoso and Hartmann, 2023). However, to the best of our knowledge, and in contrast to the situation for other countries (e.g., for Germany: Blossfeld, 1986, 1987; Stawarz, 2013, 2015; Becker and Blossfeld, 2017; for the UK: Trinh and Bukodi, 2022; for the US: Witteveen and Hossain, 2025), there is no empirical evidence for Switzerland on the impact of macro trends, such as educational expansion and skill-biased technological change, on individuals' occupational prestige across their work histories after their entry to the workforce (Sacchi et al., 2016; Häfeli et al., 2021).

Intergenerational mobility in professional prestige within modern societies is assumed to be shaped by the structural dynamics of educational expansion, long-term socio-economic trends, and short-term business cycle fluctuations (Becker et al., 2024, p. 3). Therefore, important time-dependent processes must be considered here, which affect professional careers (Mayer and Huinink, 1990). First, macro trends, such as educational expansion (changing qualifications, on the supply side of the labor market) and change in the occupational structural (change in employers' demand for qualifications) have cohort-differentiating and period-specific effects on the development of prestige over the career course (period and cohort effect) (Becker and Blossfeld, 2017). Second, long-term trends in socio-economic modernization and changes in labor market situations related to economic fluctuations have different effects on prestige in different career phases (age effect) (Blossfeld, 1986). Third, the extent to which these facts relating to age, period and cohort effects are also valid for Switzerland is a question that needs to be clarified empirically.

A study by Zangger et al. (2018) examined the influence of the aforementioned processes on labor market entry (LME) processes in Switzerland. Our study builds on this study by additionally analyzing more recent data (up to 2023). In addition, our study goes beyond the previous state of research by examining career trajectories, in addition to LME. Accordingly, based on a theoretical dynamic micro-macro model, we seek to answer the following research questions. First, does the expansion of education lead to increased chances of attaining advantageous professional positions over the course of one's career? Second, has the sectoral and occupational structural change after 1945 led to improved chances of attaining high prestige over the course of one's career across birth cohorts? Third, does socio-economic modernization lead to a collective increase in prestige over the course of one's career?

## Theoretical background

Existing studies on intragenerational prestige mobility tend to focus on the role of education (the supply side of the labor market) (i.e., human agency, referring to control over one's own actions), and do not directly measure the limited opportunities that exist (such as economic development, sectoral shifts, occupational structural change, the ecology of firms and industries, and the development of vacant positions on labor markets and unemployment) (the demand side). By contrast, the present study on intragenerational prestige mobility aims to systematically consider the interactions of both the supply and demand sides of the labor market (Becker and Blossfeld, 2017; Zangger et al., 2018; Becker et al., 2024).

Aligned with previous studies on intragenerational mobility (Becker and Blossfeld, 2017) and intergenerational mobility (Becker et al., 2024), a narrow definition of *socio-economic modernization* is used in the current analysis of prestige mobility in Switzerland (see also: Zangger et al., 2018). As suggested by Treiman (1970), the progress of modernization in different societal areas is reflected in systematic changes in industries, occupations, employment relationships, economic performance, and the degree of economic prosperity. In line with Zapf (1979), since 1945, Switzerland has seen processes of industrialization, tertiarization, technological progress, urbanization, bureaucratization, scientific rationalization of production, jobs' increasing skill requirements, mass consumption, expanded educational opportunities, increased literacy, and political democratization (Sheldon, 2005; Kriesi and Leemann, 2020). According to official statistics, in 2024, ~78% of the workforce worked in the service sector (tertiary sector), 20% in the industrial sector (secondary sector), and 2% in agriculture (primary sector). In 1860, by contrast, almost half (47%) of the workforce worked in agriculture, a slightly smaller proportion (43%) worked in industry, and 11% worked in the service sector. While in the 19th century the majority of the Swiss population lived in rural areas, by the end of 2023 around 85% of the permanent resident population in Switzerland lived in urban areas. Educational expansion in Switzerland has led to qualificational upgrading across cohorts (Buchmann et al., 1993; Becker and Zangger, 2013; Zangger and Becker, 2016). While ~19% of those born around 1950 achieved an academic degree, since the 1990s, more than 50% of women and ~40% of men have achieved this qualification (Becker and Nennstiel, 2024). These trends of modernity having a significant impact on jobs located in different industries and labor markets, and on career prospects, are also observed for Switzerland across historical periods (Buchmann and Sacchi, 1998; Oesch, 2013; Seiler, 2018; Falcon, 2020). In addition to socio-economic modernization, the development of occupational prestige across individuals' work histories is seen to be dependent on business cycles that lead to fluctuations in employment opportunities, cyclical job creation and destruction, and the ebb and flow of job openings (Zangger et al., 2018; Glauser et al., 2019).

An individual's occupational allocation can be understood as a function of their personal expectations, specifically aiming to optimize occupational prestige within the constraints of their acquired qualifications. This decision-making process occurs within particular contexts, such as entering the labor market or filling a vacant position. These individual situations, however, are themselves shaped by broader societal conditions, including trends like socio-economic modernization (e.g. skill-biased technological change and the related increase of jobs with high prestige) and prevailing labor market conditions (e.g. unemployment rate and the worsened situation of employees on unfavorable jobs).

In the course of economic development in general, and skill-biased technological change in particular, the role of general schooling, vocational education and training (VET), and tertiary education (higher VET, university training) becomes increasingly important in terms of accessing prestigious jobs with high qualificational requirements. Based on the theory of *skill-biased technological change* (e.g., Acemoglu and Autor, 2012), one would expect a rising demand for high-skilled workers and, consequently, increasing occupational prestige for those in skill-intensive occupations, over time. According to the *human capital approach* (Becker, 1962, 1975), individuals invest in their education to optimize their returns to this investment and as a result get access to favorable jobs and rewards such as income or prestige. Mincer (1974) extended the impact of formal qualifications by considering workers' informal accumulation of job- or firm-specific human capital in the course of learning-by-doing or on-the-job training. The enduring investment in their formal human capital across their work history seems to be rational if the trend of socio-economic modernization is continuously positive across periods. As their labor force experience increases, workers' prestige increases due to increasing productivity. However, due to decreasing productivity as an individual gets older (due to the effects of aging), this increase becomes smaller and smaller, so that one's status in late working life can also decrease. It is therefore assumed that there is a convex relationship between labor force experience and educational returns over the career course [*Hypothesis 1*].

The *labor queue model* proposed by Thurow (1975) stresses the interplay between the supply and demand sides of the labor market to explain social inequality at labor market entrance. According to this approach, employers rank applicants in a queue. At the front they place those applicants whose educational certificates—as assumed by the *signal and filter theory* (Spence, 1978)—indicate they can provide the appropriate level of productivity for the vacant positions. Therefore, one can assume that the applicants who are assumed to be the most productive are the ones most likely to be hired. Since employers often derive expected productivity from educational qualifications (Spence, 1978), one can therefore assume that the higher the level of education, the higher the probability of obtaining a job with greater prestige [*Hypothesis 2*].

According to the *vacancy competition model* suggested by Sørensen (1983, 1986), the employee's likelihood of achieving upward mobility in terms of increased prestige at and after their entrance into the labor market does not only depend on their qualifications, motivation, and productivity, as stressed by the human capital approach. Regardless of their level of education, employees are dependent on the development of vacant positions in their company or on other jobs offered in the labor market: “One may work hard for a promotion and not get it, because there are no promotions to be gotten. One may also work not so hard and still get a promotion because one was at the right place at the right time” (Sørensen, 1983, p. 208). Employees' general and specific qualifications are necessary to have access to a favorable vacancy, but they are not sufficient for them to be promoted. On the one hand, it is suggested that—due to the ceiling effect—the propensity to experience an increase in prestige decreases the higher the employee's position in the prestige scale. Due to the concave relationship between work experience and productivity, as suggested by economic theories, individual productivity tends to increase in the early career stages but declines later in the life course. Furthermore, there are declining opportunities for further advancement in the organizational hierarchy as one advances professionally. Therefore, it is expected that the increase in prestige decreases with the duration of labor force experience [*Hypothesis 3*]. On the other hand, an employee's likelihood of entering the labor market in a prestigious position and gaining prestige by mobility is dependent on the development of vacant positions in the course of long-term socio-economic modernization and short-term changes in labor market situations influenced by the economic business cycles (period effect) and the competition induced by fellow cohort members. One can assume that the socio-economic modernization of a society is correlated with the creation of new (more prestigious) jobs (e.g., Oesch, 2013). Therefore, a positive period effect of socio-economic modernization on workers' prestige is to be expected [*Hypothesis 4*]. However, a deterioration in economic development (e.g., indicated by rising unemployment rates) should have a negative impact on prestige at LME and make further prestige gains unlikely [*Hypothesis 5*]. In line with the vacancy competition model, there is an effect of cohort size—i.e. the absolute number of individuals who share the same birth year—on the likelihood of accessing a favorable vacancy (see: Becker et al., 2024, p. 3; Easterlin et al., 1993, p. 497). Due to the limited capacities of the labor market to absorb a larger number of potential or experienced workers, it is proposed that *a large birth cohort size* constrains the maximization of prestige, due to increased intra-cohort competition among applicants. Therefore, the greater the competition within a cohort for available jobs, the lower the prestige at LME should be, and the lower the increases in prestige over the course of a career should be [*Hypothesis 6*].

Finally, the close connection between the education system and the labor market in Switzerland, and its impact on the job and prestige prospects of graduates and employees—which can also be observed in other countries, such as Germany (Müller, 2002)—has to be considered (Buchmann and Sacchi, 1998). Due to this highly institutionalized link between these areas of the qualification-based transition system, individuals' education has a significant beneficial impact on their prestige at entry into internal firm-specific and occupation-specific labor markets, as well as across occupational careers (Blossfeld and Mayer, 1988). The trend of socio-economic modernization makes internal qualification-based labor markets increasingly important (Sousa-Poza, 2004). Therefore, one can expect that a person's educational level (in part) statistically explains the impacts of modernization on occupational prestige [*Hypothesis 7*]. This expectation is reasonable because the matching of graduates' qualifications and the qualificational requirements of their first job is institutionalized by different actors, such as labor unions, employers' associations, and the government (in Switzerland, the State Secretary of Education, Research, and Innovation). These actors define the vocational training curriculum as a reaction to occupational structural changes or in anticipation of technological changes in jobs, vocations, firms, and industries. However, it should be borne in mind that significant structural and technological changes often force young graduates and those commencing their professional careers to change their vocation in order to avoid unemployment in the early stages of their career. According to Tuma (1985), in an imperfect labor market, these misallocations can be the result of incomplete information from the employer regarding which qualifications are required as a result of changes in working processes, and incomplete information from the employee regarding their actual qualifications.

## Data

For our analyses, we use data from the *Swiss Household Panel* (SHP) (SHP Group, 2025; for more details, see: Tillmann et al., 2018, p. ix–xiii). Since 1999, households in this panel have been surveyed at regular intervals on various topics, including education and labor market participation. Since we are interested in individuals' professional careers, we use two cohorts of the SHP for our analyses: SHP I (the first survey of which took place in 1999) and SHP III (the first survey of which took place in 2013). These two cohorts are considered because respondents in these cohorts were asked to complete biographical questionnaires in the first survey wave (or one of the first survey waves), in which they were also asked about their employment history. This means that we have both retrospective data (the period before the biographical questionnaire) and prospective data (the period since panel entry) on employment history.

## Operationalization of variables

To measure the *occupational prestige* of the respondents, as the main dependent variable, we use the prestige scale suggested by Treiman (1976). To generate this, we transformed the ISCO-88 of the job into the Treiman score using the *iscogen* package (Jann, 2019). In general, the index ranges between 12 (e.g. shoe shiners) and 78 (e.g. medical doctors) (Ganzeboom and Treiman, 1996). Prestige refers to the level of status and respect associated with different types of occupations (Krueger et al., 2022). Previous studies suggest that prestige scales are useful if one is, like us, seeking to make comparisons over long historical periods (Ganzeboom et al., 1991; Wegener, 1992; Becker and Blossfeld, 2017).

**Figure 1:**
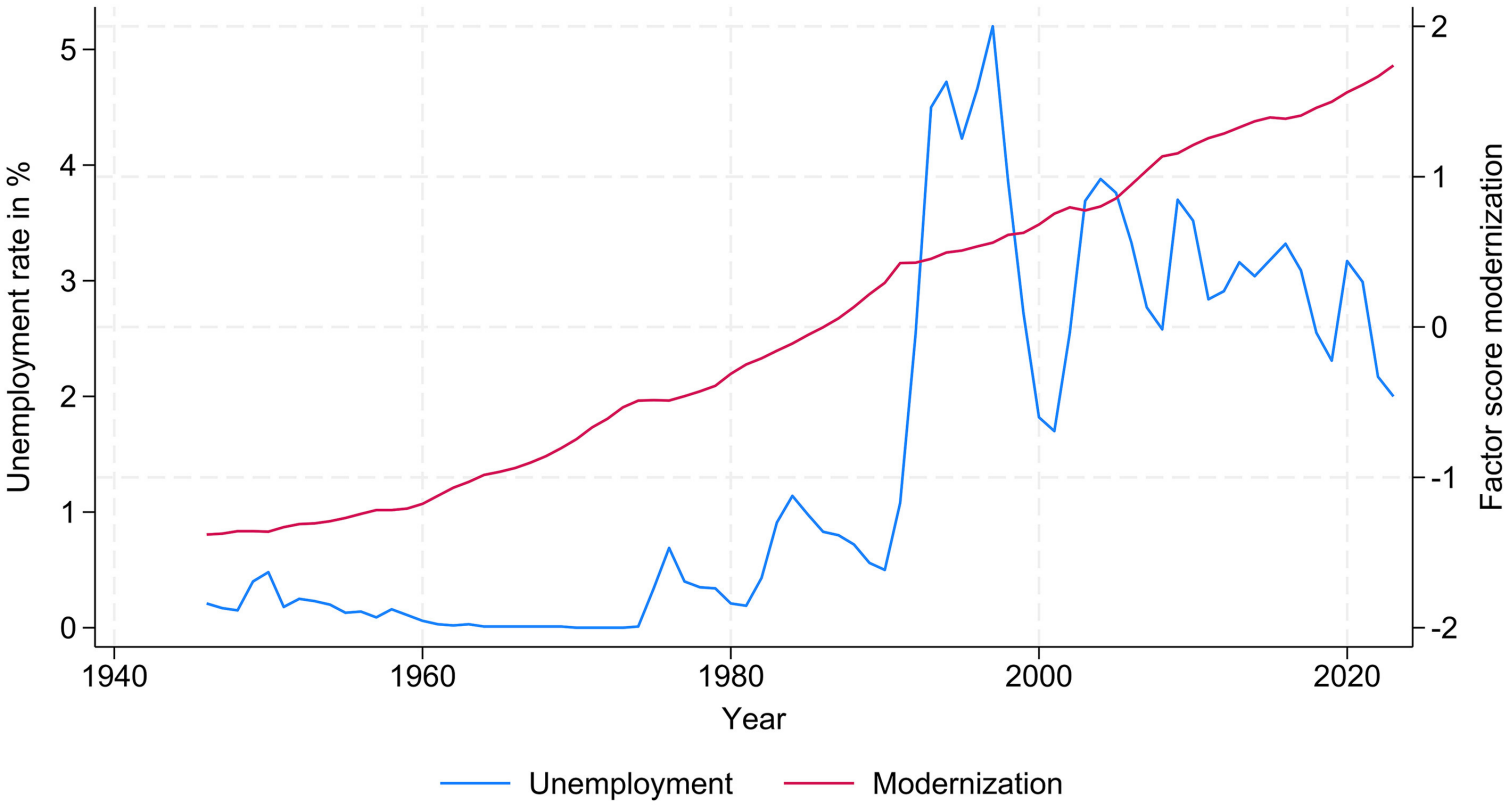
Modernization level and unemployment rate in Switzerland, 1946–2023. Source: Data from FSO; our own calculations.

*Labor market experience*, as an indicator for *age or life cycle effect*, is measured by the time since entering the labor market, and *entry into the labor market* is defined as the start of a job that lasts at least a year and which, according to the respondent, involves working hours not below 50% of a full-time position. To capture the *period effects of socio-economic change* on the outcome of individuals' careers, we use official statistics that include this information (see: Zangger et al., 2018). Based on 16 long time series for the 1946–2023 period (education spending, number of students, number of baccalaureate certificates, number of doctorates, share of employees in the tertiary sector, labor volume, real wages, consumer prices, consumer spending, population, bank staff, investments, gainfully employed, GDP, and GDP per capita), we ran an explanatory factor analysis to calculate the *modernization level* in terms of social changes in education, employment, labor market structures, occupational structures, economic business cycles, and demographic processes, as well as public, economic, and private welfare, so as to prevent the identification problem resulting from the highly correlated time series (Becker and Blossfeld, 2017, p. 119–120; Zangger et al., 2018, p. 149). The factor *modernization level* is extracted by running explanatory factor analysis, i.e. the main component method and orthogonal factor rotation. This factor explains 94% of the variance in these different time series (Kaiser-Meier-Olkin: 0.8835). Additionally, the *unemployment rate* is considered as an additional period-specific trend. Its development indicates the changing labor market conditions that might also shape individuals' career prospects. The changes in both of these period-specific indicators are documented in Figure 1 for the historical period from 1946 to 2023.

The lines in Figure 1 reflect the modernization trend and the economic business cycles (labor market conditions) throughout Switzerland's economic history in the post-war period. On the one hand, we witness a monotonic and linear trend of modernization. On the other hand, it is obvious that the development of labor market conditions has been cyclical since the 1970s, due to the strong dependency of the labor market on the business cycle, which has become increasingly dependent on the ups and downs in the long-term development of the globalized world economy. One can see the impact of the oil price shocks in the 1970s, the recession in the 1980s due to the global debt crisis, growth slumps and declines in employment in the 1990s, and the dotcom crisis and bank crashes in the 2000s.

**Figure 2:**
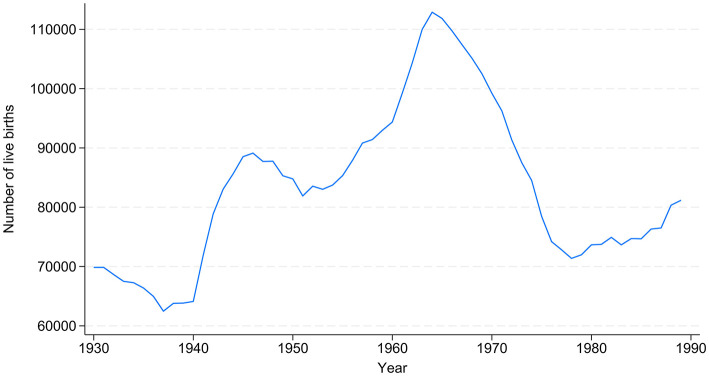
Number of live births across birth cohorts, 1930–1989. Source: Data from FSO; our own calculations.

The *number of live births per year* is used as a proxy for the *size of the birth cohorts*. At the same time, it is a proxy for the theoretically assumed *cohort effect*. We are aware that this means we cannot capture processes such as emigration and immigration, but we are convinced that this indicator measures the competition among cohort members in an adequate way (Becker et al., 2024). The development of this indicator across birth cohorts is depicted in Figure 2. We thus assign each person the number of births in their year of birth as an indicator for their cohort membership. To enable a comfortable interpretation of the coefficients, we divided this indicator by 1,000 for the regression models.

Finally, we include the number of *years of education* in our models in a time-constant manner. The highest level of education attained is considered. In the data, individuals' levels of education vary between 8 years (ISCED 1) and 21 years (ISCED 6).

## Sample selection

For 10,183 individuals from the two SHP cohorts, at least one job episode with a valid Treiman prestige score is available. We have a valid Treiman prestige score for 9,850 people, after considering only job episodes from LME onwards (333 people excluded). Of these individuals available for our analysis, we consider only job episodes that occurred in the years 1946 to 2023 (13 people excluded). Furthermore, we only consider individuals who were born after 1930 and before 1990 (686 people excluded) and who had their first *job* between the ages of 15 and 60 (172 people excluded). To reduce age-related distortions in labor market participation, we only consider job episodes that occurred between the ages of 15 and 60. Furthermore, we excluded individuals without valid information on years of education (465 people excluded). Overall, our analytical sample includes 8,514 individuals and 216,616 job-year episodes.

## Analytical strategy

To test our hypotheses, we use multilevel models to estimate linear *growth curve models* (Rabe-Hesketh and Skrondal, 2008). These models allow us to consider processes of both LME and the development of prestige over the career (for a similar approach see: Stawarz, 2013, 2015; Witteveen and Hossain, 2025). These models are suitable for our analysis because we have nested data structures (job episodes within people) whose observations are not independent of each other (Rabe-Hesketh and Skrondal, 2008; Witteveen and Hossain, 2025).

The years within individuals are used as the time unit. Thus, we assign each person a prestige value for each year in which the person had a job. If a person had several different prestige values per year, the mean value is calculated. We estimate a *two-level linear mixed regression model*. The first level represents the years and the second level the individuals. In a first step, we estimate a random intercept random slope model that includes the labor market experience, and the labor market experience squared, as well as the modernization factor, unemployment, and cohort size (Model 1). The coefficients of labor market experience and labor market experience squared are estimators for the average prestige trajectory over the career. The coefficients of the modernization factor, unemployment, and cohort size show the influence of these processes on the model intercept (i.e., occupational prestige at LME). Thus, in addition to the coefficients on labor market experience, we also obtain coefficients that show how modernization, unemployment, and cohort size affect prestige when entering the labor market. In addition to the random intercept, random slopes for the labor force experience and its squared term are included in this model for theoretical reasons, to allow for variation in the coefficients between individuals. This means that the estimates of individual occupational prestige trajectories can vary between individuals. Furthermore, the correlation between the intercept and slope of labor market experience also indicates the extent to which entry prestige and subsequent growth are related. In the next step (Model 2), education is included as an additional variable to estimate the extent to which it affects entry prestige and the extent to which the influences of period and cohort size are moderated by education. In a third step (Model 3), we estimate models that examine the extent to which modernization factor, unemployment, and cohort size effects, as well as education, affect career entry and career prestige development. To do this, we include interactions of the respective coefficients of labor market experience and labor market experience squared in the models. These interaction effects then show the influence of education, modernization factor, unemployment, and cohort size on prestige trajectories with increasing labor force experience.

**Figure 3:**
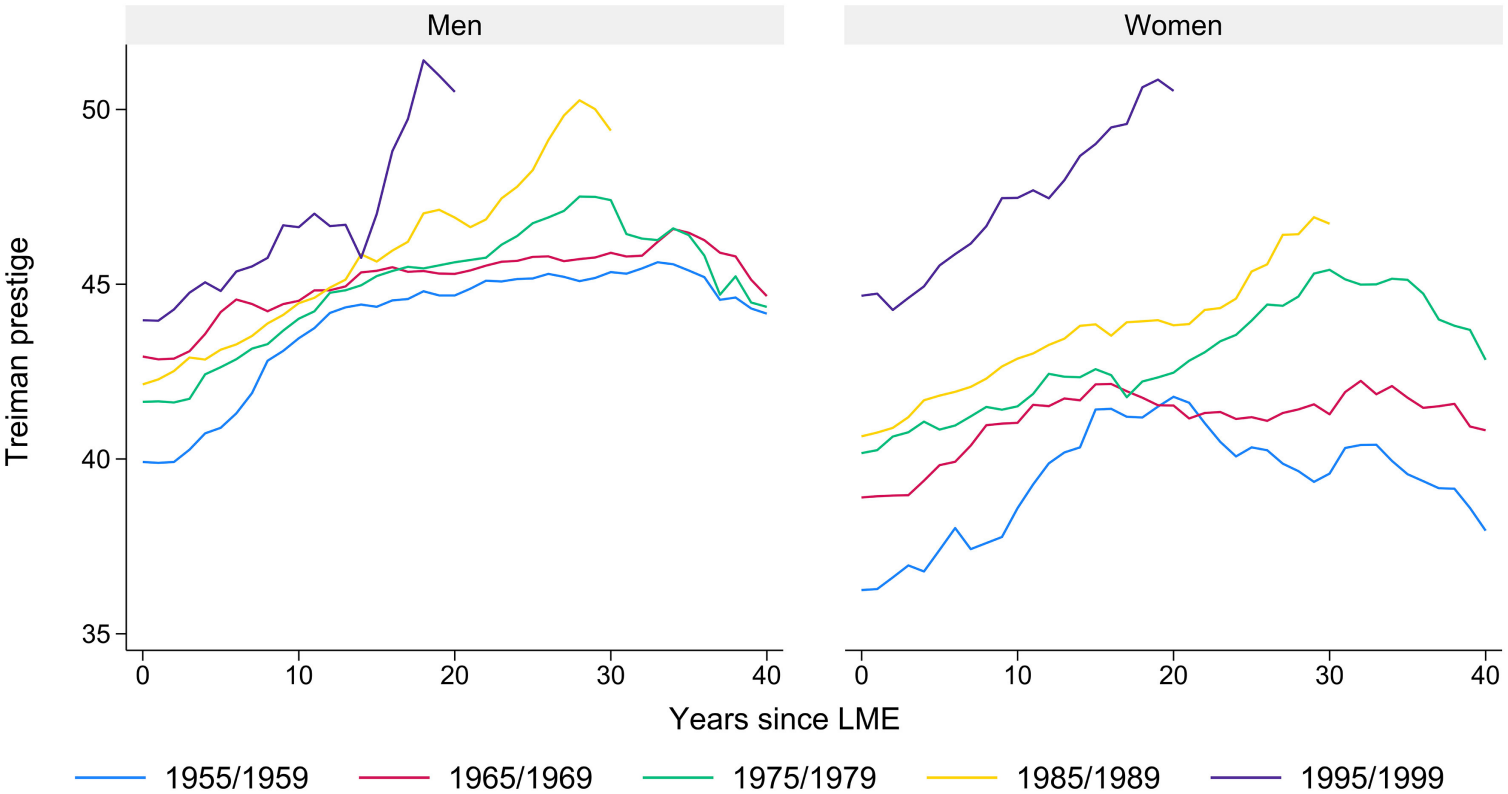
Development of average prestige across the first 40 career years for selected LME cohorts, differentiated by gender. Source: SHP; our own calculations.

The models are estimated separately for women and men since they exhibit different career patterns (Charles et al., 2001; Kriesi et al., 2010; Buchmann et al., 2010; Combet and Oesch, 2019; Häfeli et al., 2021). In regard to prestige across the career, these gender differences are depicted in Figure 3. First, the development of prestige across the work history differs between the cohorts. This is valid for both women and men. For all cohorts, higher average prestige is evident for men compared to women. Second, the younger the cohort, the higher the average prestige throughout their careers since LME. Compared to male workers, the development of prestige across birth cohorts is more pronounced for women; this obvious cohort differentiation might result from the “female” educational expansion and increased demand for qualified female workers (Oesch, 2013).

Third, the convex pattern of prestige across the career is more obvious for older cohorts than for younger cohorts. Fourth, the two younger cohorts differ in the level of average prestige among both men and, especially, women. It can be assumed that macro trends, such as occupational tertiarization and educational expansion, result in a significant increase in prestige in the later stages of their occupational careers.

## Results

The results of the growth curve models are documented in Table 1. Looking at Model 1, we see, on the one hand, the expected curvilinear relationship for both men and women, which is expressed in the positive coefficients of labor market experience and the associated negative coefficients of the squared labor market experience (this supports Hypothesis 1). Furthermore, the negative correlations between the intercept and labor market experience show that the higher the initial prestige of a job, the lower the increase in job prestige over the course of a career (this supports Hypothesis 3).

**Table 1:** Linear growth models predicting occupational prestige for men and women.

**Models**	**Men**			**Women**		
	**M1**	**M2**	**M3**	**M1**	**M2**	**M3**
**Time since LME**						
Labor force experience	0.26^***^	0.28^***^	0.36^**^	0.09^***^	0.15^***^	0.18
Labor force experience^2^	−0.00^***^	−0.00^***^	−0.01^**^	−0.00	−0.00	−0.01^*^
**Effects on intercept**						
Modernization level	0.64^***^	0.05	0.38	1.43^***^	0.12	0.32
Unemployment rate	−0.05^***^	−0.05^***^	−0.08^*^	−0.08^***^	−0.08^***^	0.01
Cohort size	−0.02	−0.04^***^	−0.04^***^	0.02	−0.02	−0.01
Years of education		2.01^***^	1.92^***^		1.93^***^	1.74^***^
**Effects on slope**						
Modernization level × labor force experience			−0.05^**^			−0.02
Modernization level × labor force experience^2^			0.00^***^			−0.00
Unemployment rate × labor force experience			−0.00			−0.01^**^
Unemployment rate × labor force experience^2^			0.00			0.00^***^
Cohort size × labor force experience			−0.00			−0.00^**^
Cohort size × labor force experience^2^			0.00			0.00^***^
Years of education × labor force experience			0.00			0.02^***^
Years of education × labor force experience^2^			0.00			−0.00
Intercept	43.79^***^	16.67^***^	18.04^***^	39.71^***^	17.19^***^	18.78^***^
**Variance components**						
var(Labor force experience)	1.06^*^	1.06^*^	1.06^*^	1.16^***^	1.16^***^	1.15^***^
var(Labor force experience^2^)	0.00^***^	0.00^***^	0.00^***^	0.00^***^	0.00^***^	0.00^***^
var(Intercept)	139.66^***^	102.01^***^	102.06^***^	125.07^***^	98.12^***^	97.74^***^
corr(Labor force experience, Labor force experience^2^)	−0.91^***^	−0.91^***^	−0.91^***^	−0.92^***^	−0.92^***^	−0.92^***^
corr(Labor force experience, Intercept)	−0.41^***^	−0.49^***^	−0.49^***^	−0.39^***^	−0.48^***^	−0.48^***^
corr(Labor force experience^2^, Intercept)	0.27^***^	0.30^***^	0.30^***^	0.26^***^	0.31^***^	0.31^***^
var(Residual)	14.60^***^	14.60^***^	14.59^***^	16.95^***^	16.94^***^	16.94^***^
*Observations*	117,730	117,730	117,730	98,886	98,886	98,886
*N*	4,093	4,093	4,093	4,421	4,421	4,421
*AIC*	694,313	692,379	692,308	602,051	600,274	600,216

^***^p < 0.001; ^**^p < 0.01; ^*^p < 0.05.

Source: SHP; our own calculations.

For both men and women, there is a significant positive influence of modernization on career entry prestige (this supports Hypothesis 4) and a significant negative influence of unemployment on career entry prestige (this supports Hypothesis 5). For men and women alike, there no statistically significant influence of cohort size on career entry prestige (contrary to Hypothesis 6). In line with Hypothesis 7, Model 2 shows that, once education is controlled for, the influence of modernization on job entry prestige is no longer statistically significant, for both men and women. Interestingly, compared to Model 1 the expected negative influence of cohort size on occupational prestige also becomes obvious for men. For both men and women, it is found that the more years of education, the higher the occupational prestige at LME (supporting Hypothesis 2).

Finally, considering the influence of societal macro trends on prestige trajectories (Model 3), the following findings are revealed. The trend of modernization has no significant influence on the increase in women's occupational prestige. For men, however, there is a slightly negative influence that decreases with increasing work experience. This could indicate that if male graduates enter the labor market in times of high modernization, these young professionals generally have access to jobs with a higher level of prestige and therefore cannot achieve any further increases, due to ceiling effects in the first years of their careers. Overall, these findings tend to contradict Hypothesis 4.

In contrast to the relationship expected in Hypothesis 5, no statistically significant influence of the unemployment rate on the development of prestige is found for men across the observed periods. For women, however, it is found that there is a negative influence of worse labor market conditions in periods of pronounced unemployment. At the beginning of their working lives, in particular, unfavorable labor market conditions have a negative impact on increases in prestige. This could indicate different mobility processes for men and women in the course of starting a family and the subsequent interruptions in employment, such that once men are in employment, they are better protected from the negative effects of unemployment due to their more continuous employment history.

In line with Hypothesis 6—though only for women—it is found that more competition from more cohort members, especially at the beginning of one's working life, is associated with lower increases in prestige. Furthermore, again only statistically significant for women—it is true that the more years of education a person has, the higher the increase in prestige, especially at the beginning of their working life.

## Discussion of the findings

To consider the particular effects of social dynamics in Swiss society on women's and men's career outcomes, in terms of occupational prestige, the impact of macro trends—such as modernization level, unemployment rate, and cohort size located at the macro level—on prestige at the individual level has been analyzed in a dynamic micro-macro analysis. Additionally, the specification of the models considers age, period, and cohort effects on the development of prestige across work histories simultaneously. At first glance, the age effects of labor market exposure, the period effects of modernization and labor market conditions, and the cohort effects of demographic processes on individuals' career outcomes across different historical periods and stages in individuals' work histories are in line with the hypotheses derived from different sociological and economic labor market theories.

Things change when the individual's education and training is considered within the wider context of the simultaneously ongoing educational expansion. First, it is found that the effect of modernization becomes statistically insignificant. Against the background of the “race between education and technology”, it is argued that increased investment in human capital becomes increasingly important to optimize career outcomes in terms of prestige. In the Swiss case, in particular, this might be true for prestige mobility in the occupation-specific labor market of small and medium-sized companies. Second, at the same time, it is found that intra-cohort competition for vacancies increases in periods characterized by high levels of modernity, when there is a high supply of well-qualified manpower. Given the availability of vacant jobs, therefore, this interrelation results in the negative impact of cohort size on career outcomes. Third, considering the time-dependent dynamic of individuals' careers, this process is only valid for men. For women, it seems to be true that investing in human capital neutralizes the structural process we observe at the societal level. Compared to women, the complex processes of economic modernization, as well as the related socio-economic changes in the labor market, have a particularly strong impact on male workers' development of prestige over the course of their careers.

## Limitations

Our study has several limitations that indicate possible avenues for future research. First, our approach to modeling modernization relies heavily on economic indicators and educational structures, due to the requirements of long time series data. As a result, broader societal dimensions, such as shifts in family structures, cultural norms, or value systems, are not explicitly considered, despite their documented relevance in long-term social change processes (Levy, 2013). Second, we are not able to incorporate migration-related population changes (Fellini and Guetto, 2019), even though immigration has played a significant role in shaping the Swiss labor market and occupational structure, particularly in recent decades. Third, while we analyze data separately for men and women, our study does not follow an intersectional framework. Some of our findings indicate gender-specific patterns in the determinants of occupational prestige, suggesting that future research could benefit from a more systematic exploration of how gender intersects with other axes of inequality (Combet and Oesch, 2019). Fourth, our modeling strategy adopts a multilevel approach, as is common in intergenerational mobility research (e.g., Stawarz, 2013, 2015; Witteveen and Hossain, 2025) and does not apply more causal modeling techniques, such as longitudinal structural equation models (Little, 2024). Such approaches could be valuable for capturing potential bidirectional relationships between education, labor market experiences, and occupational prestige. These limitations highlight fruitful directions for future research to refine and extend the framework proposed in this study.

## Summary and conclusion

Against the background of previous and current research on social stratification and mobility in Switzerland (e.g., Falcon, 2020), the goal of our study was to reveal the impact of structural change on career outcomes, in terms of occupational prestige, in a dynamic micro-macro model of the development of occupational prestige across women's and men's work histories in different birth cohorts. Following on from a previous study of LME in Switzerland (Zangger et al., 2018), the aim of our study was to contribute to a better understanding of how the long-term modernization process and changes in labor market conditions in the course of the business cycle have affected career outcomes, in terms of occupational prestige, since 1946. To the best of our knowledge, this is the first analysis on the time dependency of individuals' job rewards across their careers after LME, as well as across different periods and cohorts, in Switzerland.

To achieve the aforementioned aim, we combined longitudinal data on occupational careers based on the SHP with macro data based on the long time series collected by the Swiss Office of Statistics. This dataset covers the period between 1946 and 2023. It was analyzed by estimating growth curve models in a time-related multilevel design. The main reason for applying this statistical procedure is the proven argument that workers' access and allocation to jobs that provide a favorable level of prestige depend not only on workers' education and labor force experience but also on the availability of vacancies in firms and industries. Since the number of vacant jobs is the result of complex processes of socio-economic modernization, the distribution of prestige across work histories might vary across birth cohorts and historical periods.

As a first result, the study revealed for Switzerland that workers' benefits in terms of prestige have increased across the LME cohorts and across their work histories. Different cohorts are the cultural carriers of societal changes, such as educational expansion and tertiarization of jobs. In particular, younger cohorts have profited from these structural changes, and this is true for both women and men. As a second finding, it becomes obvious that there are different time-related effects, at both the individual level and the structural level, in regard to workers' career outcomes. To start with the individual level, semi-dynamic theoretical approaches, such as human capital theory, are supported empirically. Investment in general education and vocational training—that is to say, skills and credentials—is essential to optimize returns to education. In the course of tertiarization and the increased qualificational requirements of favorable jobs, investment in tertiary education became increasingly necessary. As their labor force experience increases, both women's and men's level of prestige develops in a convex function. A third finding is the positive impact of the modernization trend, the negative effect of worse labor market conditions, and the negative effect of cohort size on increases in prestige across individuals' careers. While, in the period under review, the level of prestige increased simultaneously with the linear modernization trend, the increase in the unemployment rate since the 1970s, as well as intra-cohort competition, had negative effects on prestige as a reward for achievements in individuals' work histories. This development was particularly true for male workers, while women were more likely to be able to compensate for the negative effects of macro trends by investing in their education. Overall, investment in general schooling and vocational training, as well as in tertiary education, has outpaced the adverse impacts of structural changes in the economic system and related labor markets.

Regarding the questions we raised at the beginning, we can give the following answers. There is empirical evidence that, during the period under review, the expansion of education, which began timidly in the 1970s and has accelerated since the 1990s, led to increased chances of attaining advantageous professional positions over the course of individuals' careers, measured in terms of prestige as the return on education. It is obvious that sectoral and occupational structural changes after 1945 led to improved chances of attaining high prestige over the course of individuals' careers for the younger birth cohorts. Finally, it was revealed that socio-economic modernization, with the creation of new vacancies with advanced qualification requirements and higher prestige, led to a collective increase in prestige across successive cohorts and over the course of women's and men's careers. In particular, younger birth cohorts profited from intertwined macro trends, such as educational expansion and the socio-economic modernization, i.e. from the “race between education, technology and tertiarization”.

In theoretical and methodological respects, it has to be stressed that, although the interplay between structural and individual characteristics in determining access to favorable job positions has long been a focus of research in the areas of social stratification and mobility, this approach has not previously been applied to the Swiss case. Despite the suboptimal situation with regard to data, an innovative theoretical and methodological framework has been outlined here that could offer a promising way to describe and explain the social dynamics of the Swiss social structure. Longitudinal data analysis in a multidimensional age-period-cohort design should be extended in the current research on social change of societies, their social structures, organizations, and institutions. In future mobility research, there is a need to combine individuals' resources and motivation with respect to job mobility with the objective structural constraints of labor markets and workers' subjective assessments of the development of jobs, vocations, and rewards (Mayer et al., 2010).

## Data Availability

Publicly available datasets were analyzed in this study. This data can be found here: https://www.swissubase.ch/en/catalogue/studies/6097/20679/overview. The replication material for this study can be found here: https://osf.io/2ca74.
